# The impact of the COVID-19 pandemic on the diagnosis, stage, and treatment of esophagogastric cancer

**DOI:** 10.1007/s00535-023-02009-3

**Published:** 2023-07-31

**Authors:** Benthe H. Doeve, Jeanne A. C. Bakx, Peter D. Siersema, Camiel Rosman, Nicole C. T. van Grieken, Mark I. van Berge Henegouwen, Johanna W. van Sandick, Marcel Verheij, Maarten F. Bijlsma, Rob H. A. Verhoeven, Hanneke W. M. van Laarhoven

**Affiliations:** 1 Department of Medical Oncology, Amsterdam UMC, Location University of Amsterdam, Meibergdreef 9, De Boelelaan 1118, 1081 HZ Amsterdam, The Netherlands; 2 Cancer Center Amsterdam, Cancer Treatment and Quality of Life, Amsterdam, The Netherlands; 3 Center for Experimental and Molecular Medicine, Laboratory for Experimental Oncology and Radiobiology, Amsterdam UMC, University of Amsterdam, Meibergdreef 9, Amsterdam, The Netherlands; 4 Oncode Institute, Meibergdreef 9, Amsterdam, The Netherlands; 5 Department of Research and Development, Netherlands Comprehensive Cancer Organisation (IKNL), Utrecht, The Netherlands; 6 Department of Gastroenterology and Hepatology, Erasmus MC University Medical Center, Rotterdam, The Netherlands; 7 Department of Surgery, Radboud University Medical Center, Nijmegen, 6500 HB The Netherlands; 8 Department of Pathology, Amsterdam UMC, Location Vrije Universteit Amsterdam, De Boelelaan 1117, Amsterdam, The Netherlands; 9 Department of Surgery, Amsterdam UMC, Location University of Amsterdam, Meibergdreef 9, Amsterdam, The Netherlands; 10 Department of Surgery, Antoni Van Leeuwenhoek Hospital - Netherlands Cancer Institute, Amsterdam, The Netherlands; 11 Department of Radiation Oncology, Radboud University Medical Center, Nijmegen, 6500 HB The Netherlands

**Keywords:** COVID-19, Esophageal cancer, Gastric cancer, Incidence, Treatment

## Abstract

**Background:**

The COVID-19 pandemic has affected the entire global healthcare system, including oncological care. This study investigated the effects of the COVID-19 pandemic on the diagnosis, stage, and treatment of esophagogastric cancer in the Netherlands.

**Methods:**

Patients diagnosed in 2020 were divided into 5 periods, based on the severity of the COVID-19 pandemic in the Netherlands, and compared to patients diagnosed in the same period in the years 2017–2019. Patient characteristics and treatments were evaluated for esophageal cancer (EC) and gastric cancer (GC) separately.

**Results:**

The number of esophagogastric cancer diagnoses decreased prominently during the first 2 months of the COVID-19 pandemic. During this period, a significantly higher percentage of GC patients was diagnosed with incurable disease (52.5% in 2017–2019 and 67.7% in 2020, p = 0.011). We observed a significant reduction in the percentage of patients with potentially curable EC treated with resection and neoadjuvant chemoradiotherapy (from 35.0% in 2017–2019 to 27.3% in 2020, p < 0.001). Also, patients diagnosed with incurable GC were treated less frequently with a resection (from 4.6% in 2017–2019 to 1.5% in 2020, p = 0.009) in the second half of 2020.

**Conclusions:**

Compared to previous years, the number of esophagogastric cancer diagnoses decreased in the first 2 months of the COVID-19 pandemic, while an increased percentage of patients was diagnosed with incurable disease. Both in the curative and palliative setting, patients were less likely to be treated with a surgical resection.

**Supplementary Information:**

The online version contains supplementary material available at 10.1007/s00535-023-02009-3.

## Introduction

Since December 2019, the Coronavirus Disease 2019 (COVID-19) pandemic caused by the SARS-CoV-2 virus has greatly impacted health care systems around the world. Many resources were preferentially dedicated to COVID-19 patients, forcing clinicians to make difficult triage decisions. In the Netherlands, the first COVID-19 patient was diagnosed on February 27, 2020 [1]. Subsequently, the Dutch government introduced measures to contain the virus spread and to protect its most vulnerable inhabitants [1]. From the second week of March 2020, these measures included social distancing, working from home, and closing schools and restaurants [2]. As in the rest of the world, the surge in COVID-19 patients increased pressure on standard care in the Netherlands. This resulted in major downscaling of standard care, including oncological care, especially during the first outbreak [3]. In April 2020, a significant decrease in the number of cancer diagnoses was observed [4–9]. This decrease was most likely caused by a combination of patient delay and doctor delay [4]. Patient delay occurs when an individual with symptoms postpones seeking medical attention, while doctor delay occurs when symptoms are not diagnosed timely. The latter can be due to postponed physical examinations as consultations were replaced by telehealth consultations, reduced diagnostic evaluation capacities, or halted diagnostic endoscopy programs, all of which occurred during the COVID-19 pandemic.

A diagnostic delay is of particular concern in cancers of the esophagus, esophagogastric junction, and stomach, together also known also as esophagogastric cancer. Esophagogastric cancer has a poor prognosis and accounted for 13.2% of all cancer deaths worldwide in 2020 [10]. Presenting symptoms of this rapidly progressive cancer type include fatigue, dysphagia, and weight loss [11]. Population screening esophagogastroduodenoscopy is not performed in the Netherlands, but screening is sporadically considered in men with chronic gastroesophageal reflux and two more risk factors, such as smoking or central obesity. In addition, screening is recommended every 3–5 years in patients with Barrett’s esophagus. Otherwise, patients are generally referred to a gastroenterologist for an endoscopy based on symptoms, such as anemia, weight loss, or dysphagia [12]. Diagnostic delay in esophagogastric cancer could result in more advanced disease at the time of diagnosis and the associated weight loss may also lead to poor tolerance of treatment with higher chances of complications. For instance, recent research has shown that underweight patients are more prone to develop postoperative complications after esophagectomy [13]. Downscaling during the COVID-19 pandemic may have resulted in postponed or different, potentially suboptimal, treatment options for patients with esophagogastric cancer. Altogether, the COVID-19 pandemic may have had a major impact on the care of patients with esophagogastric cancer. This study aimed to investigate the effects of the COVID-19 pandemic in 2020 on the diagnosis, stage, and type of treatment of esophagogastric cancer in the Netherlands.

## Methods

### Study population

For inclusion, patients diagnosed with esophageal cancer (EC) or gastric cancer (GC) between January 1, 2017 and December 31, 2020 were selected from the Netherlands Cancer Registry (NCR). The NCR is a nationwide population-based cancer registry that covers the entire Dutch population of more than 17 million people. The NCR is directly linked to the national pathological archive (PALGA) wherein all histologically confirmed cancer diagnoses are registered [14]. Every pathology laboratory in the Netherlands is part of PALGA, and all pathological reports are automatically transferred to the central PALGA database. Trained data managers routinely extract information on diagnosis, patient, tumor, and treatment characteristics from electronic medical records in the hospitals. Patients treated in foreign hospitals were not included for this study as the NCR only registers information from Dutch hospitals. This study included patients with all types of adenocarcinomas of the stomach and the esophagus, and all types of squamous cell carcinomas of the esophagus. Carcinomas not otherwise specified were also included, but patients with a histological diagnosis of a neuro-endocrine tumor or neuro-endocrine carcinoma, lymphoma, GIST, melanoma, or sarcoma were excluded.

### Definitions

The year 2020 was divided into 5 periods based on the severity of the COVID-19 pandemic in the Netherlands. Period 1 spanned weeks 1–8 (i.e., before the COVID-19 pandemic); period 2 included weeks 9–12 (after the first COVID-19 case and before the lockdown); period 3 week 13–17 (during lockdown); period 4 weeks 18–26 (after lockdown and during scaling back up hospital care); and period 5 encompassed weeks 27–52 (stabilizing hospital care). Patients were categorized into one of the five time periods based on the date of diagnosis for all analyses.

Esophagogastric cancer was classified into early carcinoma (cT1A or 1B, cN0 or cNX and cM0); potentially curable disease (cT1N + , cT2, cT3, cT4A, or cTX and cM0), palliative disease (cT4B or cM1), and unknown disease severity according to the TNM staging system for analysis of changes in disease stage [15]. Patients were classified in the EC or GC group based on the tumor location originally provided by PALGA. Cardia and junction tumors were classified as EC or GC based on treatment. Patients who underwent gastric resection were classified as GC, whereas patients who underwent esophageal resection and/or received chemoradiotherapy (CRT) and/or received any other treatments were grouped as EC.

In the Netherlands, treatment of esophagogastric cancer is selected based on the guidelines of the Dutch Federation of Medical Specialists [16, 17]. For patients with potentially curable EC, treatment selection is based on tumor stage for potential endoscopic resection, performance status, resectability of the tumor, operability of the patient, and patient preference [16]. Treatments were defined as follows; (1) neoadjuvant chemoradiotherapy with esophageal resection (nCRT-ER), in which patients were treated with chemotherapy and at least 37.8 Gy fractionated radiotherapy followed by resection, (2) definitive chemoradiotherapy (dCRT), in which patients were treated with chemotherapy and more than 41.4 Gy radiotherapy not followed by resection, (3) neoadjuvant chemoradiotherapy without resection (nCRT), in which patients were treated with chemotherapy and 37.8–41.4 Gy radiotherapy not followed by resection, (4) esophageal resection without neoadjuvant chemoradiotherapy (ER), and (5) other treatments.

For patients with potentially curable GC, treatment selection is based on tumor stage, performance status, operability of the patient, and patient preference [17]. Treatments were defined as follows; (1) chemotherapy with gastric resection (CT-GR), in which patients were treated with either neoadjuvant and/or adjuvant chemotherapy of any dosage combined with resection, (2) gastric resection without chemotherapy (GR), (3) chemotherapy without resection (CT), and (4) other treatments.

Survival in patients with incurable disease and a proper performance status can be improved with chemotherapy combined with targeted therapy. Surgery, short-course radiotherapy or placement of stents may be considered to palliate symptoms [16, 17]. Patients with incurable disease were classified into groups based on the most invasive treatment. Therefore, patients were classified either in the group undergoing resection with or without other treatment, the group undergoing chemotherapy (CT) with or without non-concomitant radiotherapy to alleviate symptoms, the group undergoing solely radiotherapy (RT) to alleviate symptoms, or the group undergoing other treatments such as best supportive care.

Time to start treatment was defined as the time from diagnosis until the start of any treatment indicated, including chemo (radio) therapy and resection. The time until direct resection was defined as the time from diagnosis until primary surgery in patients without any (neo) adjuvant treatment. The time until surgery was defined as the time between the end of neoadjuvant treatment and resection.

### Statistical analysis

Comparisons were performed between the five periods in 2020 and the corresponding periods in 2017–2019. Tumor characteristics and treatments were displayed as counts and percentages and chi-squared tests were used to compare the percentages between groups. Treatments were compared separately for the potentially curable setting and the palliative setting for both EC and GC. The time between diagnosis, neoadjuvant treatment, and resection was represented as the mean with the standard deviation (SD). Times were assessed separately for the potentially curable and the palliative setting for both EC and GC. Two-sided unpaired Wilcoxon tests were used for comparison between 2017 and 2019 and 2020 for all periods. Two-year overall survival was analyzed using Cox regression per group (potentially curable EC, palliative EC, potentially curable GC, and palliative GC) and correcting for sex, age, comorbidities, and performance status. Survival data was censored after 731 days to ensure that the follow-up data of all patients were comparable. A *p* < 0.05 was considered statistically significant. All analyses were performed using R version 4.1.1 software [18].

## Results

### Baseline characteristics

In total, 15,715 patients were included in this study, of whom 9,959 EC patients and 5,756 GC patients (Table 1*).* Age, histology, stage, and comorbidities were evenly distributed between patients diagnosed in 2020 and patients diagnosed in 2017–2019. In 2020, there were significantly more female EC patients (*p* = 0.05) and significantly more GC patients had a worse performance status (*p* = 0.03). Additional patient characteristics for all periods separately are shown in *Online Resource 1*.

**Table 1:** Baseline characteristics of 15.715 included patients with esophageal or gastric cancer

	Esophageal cancer			Gastric cancer		
	2017–2019	2020	p-value	2017–2019	2020	p-value
Patients	7568	2391		4325	1431	
Sex						
Female	1945 (25.7)	664 (27.8)	0.048	1549 (35.8)	515 (36.0)	0.931
Male	5623 (74.3)	1727 (72.2)		2776 (64.2)	916 (64.0)	
Age						
< 60 years	1208 (16.0)	367 (15.3)	0.715	742 (17.2)	272 (19.0)	0.203
60–74 years	3978 (52.6)	1256 (52.5)		1678 (38.8)	527 (36.8)	
> 74 years	2382 (31.5)	768 (32.1)		1905 (44.0)	632 (44.2)	
Histology						
Adenocarcinoma	5542 (73.2)	1738 (72.7)	0.866	4281 (99.0)	1410 (98.5)	0.210
Squamous cell carcinoma	1966 (26.0)	633 (26.5)		0 (0.0)	0 (0.0)	
Other	60 (0.8)	20 (0.8)		44 (1.0)	21 (1.5)	
Stage						
Early carcinoma	207 (2.7)	67 (2.8)	0.119	94 (2.2)	32 (2.2)	0.268
Potentially curable	4783 (63.2)	1462 (61.1)		1981 (45.8)	621 (43.4)	
Incurable	2572 (34.0)	857 (35.8)		2246 (51.9)	778 (54.4)	
Unknown	6 (0.1)	5 (0.2)		4 (0.1)	0 (0.0)	
Comorbidities						
0	3203 (44.6)	1056 (45.0)	0.818	1790 (44.1)	625 (44.3)	0.844
1	2360 (32.8)	755 (32.1)		1326 (32.7)	468 (33.2)	
2 or more	1623 (22.6)	538 (22.9)		945 (23.3)	318 (22.5)	
Performance status						
ECOG 0	2222 (38.8)	741 (38.2)	0.057	958 (34.0)	374 (35.8)	0.031
ECOG 1	2369 (41.4)	764 (39.4)		1129 (40.1)	366 (35.1)	
ECOG 2	745 (13.0)	273 (14.1)		428 (15.2)	173 (16.6)	
ECOG 3 or 4	385 (6.7)	160 (8.3)		302 (10.7)	131 (12.5)	

All data are represented as n (%). Counts for 2017–2019 are shown as the sum of the 3 years. Percentages for 2017–2019 are shown as the average of the 3 years

### Incidence

**Fig. 1:**
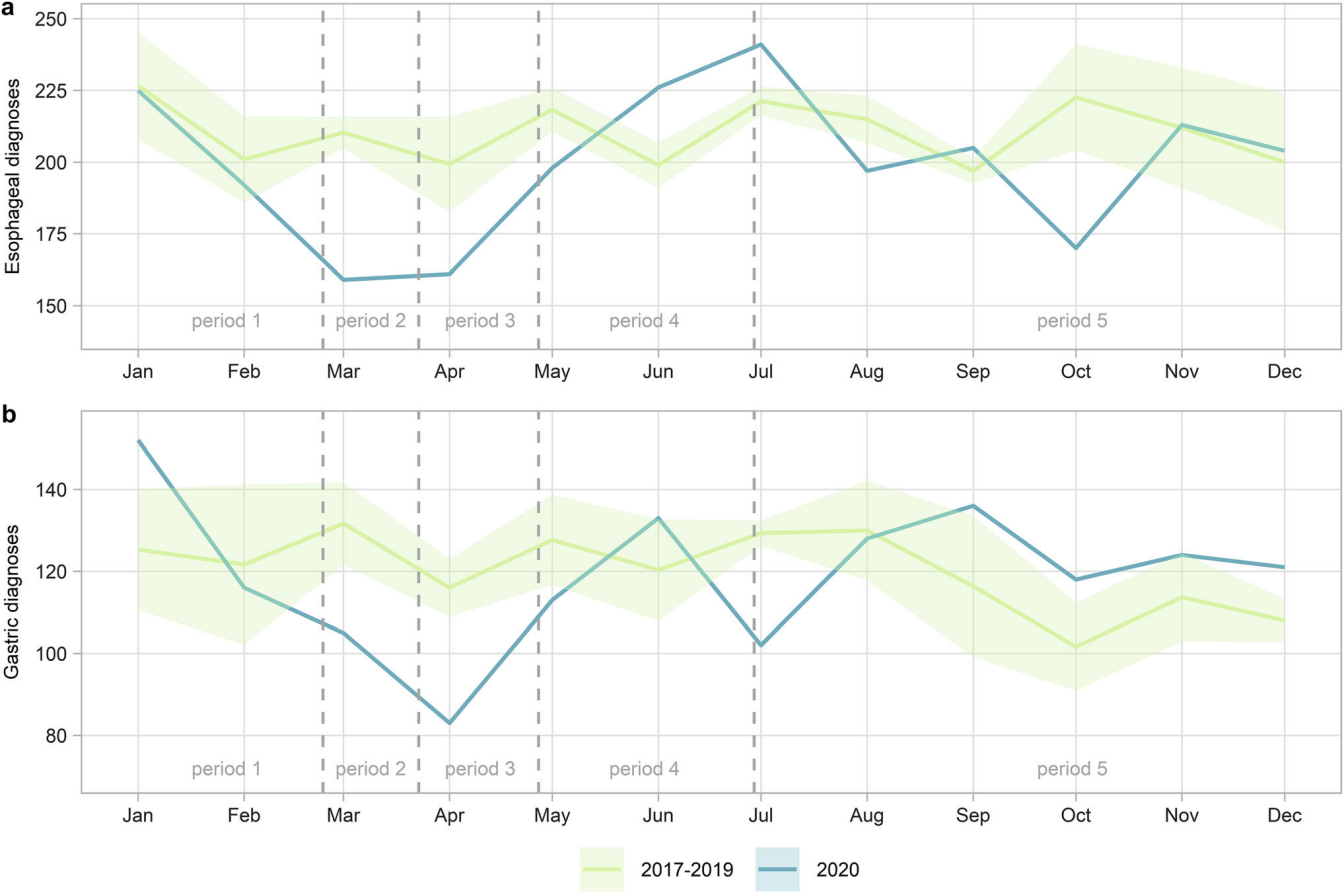
Plot of the number of diagnoses in each month for the year 2020 compared to 2017–2019 where **a** shows the number of esophageal cancer diagnoses and **b** shows the number of gastric cancer diagnoses

**Fig. 2:**
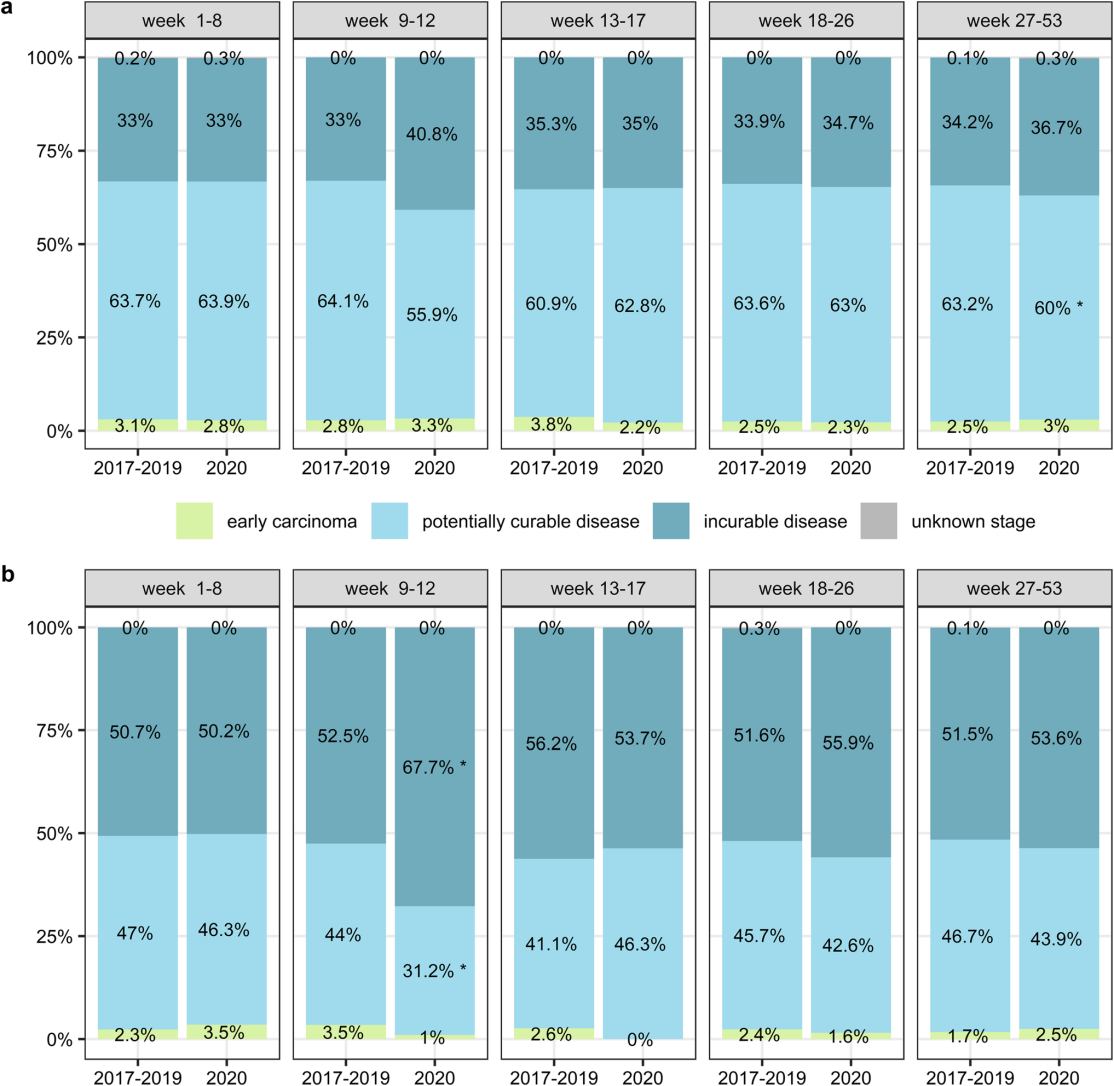
Plot of the distribution of cancer stages stratified by the period of diagnosis where **a** shows the different stages of esophageal tumors and **b** shows the different stages of gastric tumors

The absolute number of diagnoses decreased most prominently in the periods before and during the social lockdown (Fig. 1). The total incidence of EC in 2020 decreased with 5.2% compared to previous years (*n* = 2523 average in 2017–2019 and *n* = 2391 in 2020), whereas the incidence of GC diagnoses remained stable (*n* = 1442 average in 2017–2019 and *n* = 1431 in 2020). When analyzing the five separate periods, the percentage of GC patients diagnosed with incurable disease increased significantly (from 52.5% in 2017–2019 to 67.7% in 2020, *p* = 0.01) after the first COVID-19 patient was diagnosed in the Netherlands (Fig. 2). In the same period, we also observed a relative but not significant increase in EC patients diagnosed with incurable disease (from 33.0% in 2017–2019 to 40.8% in 2020, *p* = 0.09). No significant difference was found in the distribution of stages of disease at time of diagnosis over the entire year 2020 compared to 2017–2019.

### Treatment

**Fig. 3:**
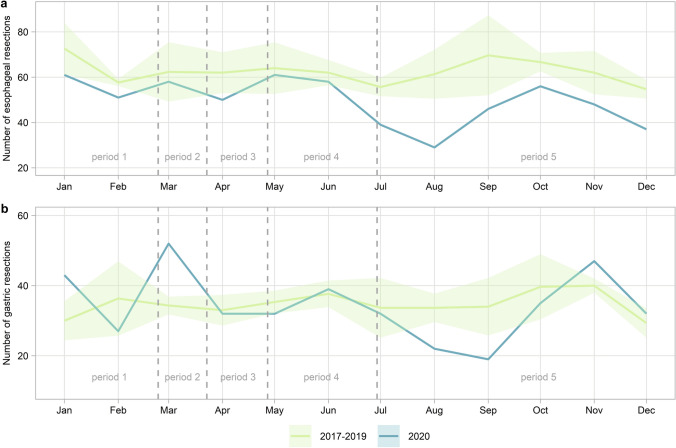
Plot of the number of resections in each month where **a** shows the number of esophageal resections and **b** shows the number of gastric resections

**Fig. 4:**
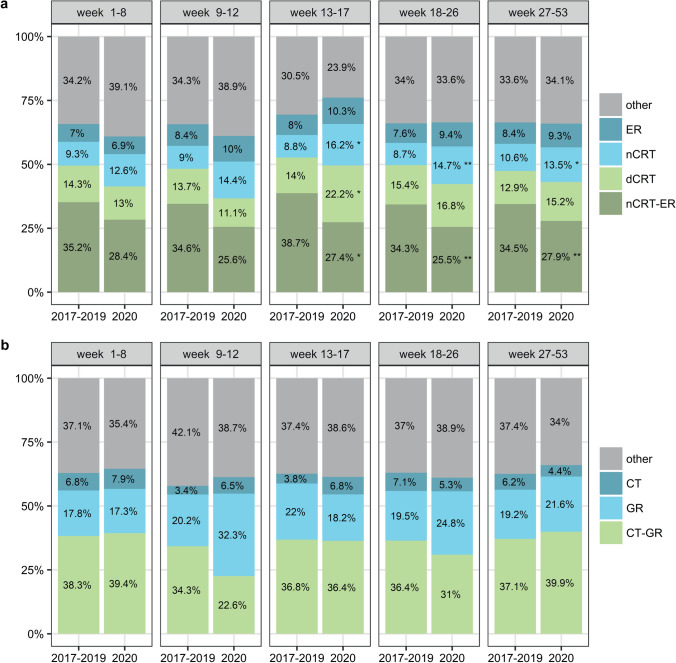
Plot of the overview of treatments given in the curable setting stratified by period of diagnosis where **a** shows the treatments of patients with potentially curable esophageal cancer and **b** shows the treatments in patients with potentially curable gastric cancer. nCRT-ER; neoadjuvant chemoradiotherapy with esophageal resection, dCRT; definitive chemoradiotherapy, nCRT; neoadjuvant chemoradiotherapy without resection, ER; esophageal resection without neoadjuvant chemoradiotherapy, CT-GR; gastric resection with (neo)adjuvant chemotherapy, GR; gastric resection without chemotherapy, CT; chemotherapy without resection

During the second half of 2020, a profound decrease in the number of resections was seen (Fig. 3). Over the entire year of 2020, we observed a significant decrease in the percentage of potentially curable EC patients treated with nCRT-ER (from 35.0% in 2017–2019 to 27.3% in 2020, *p* < 0.001) and a significant increase in the percentage of patients treated only nCRT (from 9.8% in 2017–2019 to 13.9% in 2020, *p* < 0.001). The difference in nCRT-ER and nCRT was most prominent in patients diagnosed during and after the lockdown (period 3, 4 and 5, Fig. 4a). At the same time, we found a significant increase in the use of dCRT during the lockdown (period 3, 14.0% in 2017–2019 compared to 22.2% in 2020, *p* = 0.04). This increase was seen both in patients with histologically diagnosed esophageal squamous cell carcinomas and adenocarcinomas (28.8% in 2017–2019 compared to 35.7% in 2020 and 8.6% in 2017–2019 and 14.7% in 2020 respectively). No difference in the percentage of patients treated with dCRT was observed over the whole year of 2020 compared to 2017–2019 (*p* = 0.10). For the patients with potentially curable GC, there was no significant difference in treatment over the entire year. However, a similar decreasing trend in CT-GR was observed after the first COVID-19 patient was diagnosed in the Netherlands (period 2, 34.3% in 2017–2019 compared to 22.6% in 2020, p = 0.209) with a concurrent increase in CT or GR alone (Fig. 4b). This concurrent increase is also evident by the peak of gastric resections in March (Fig. 3).

**Fig. 5:**
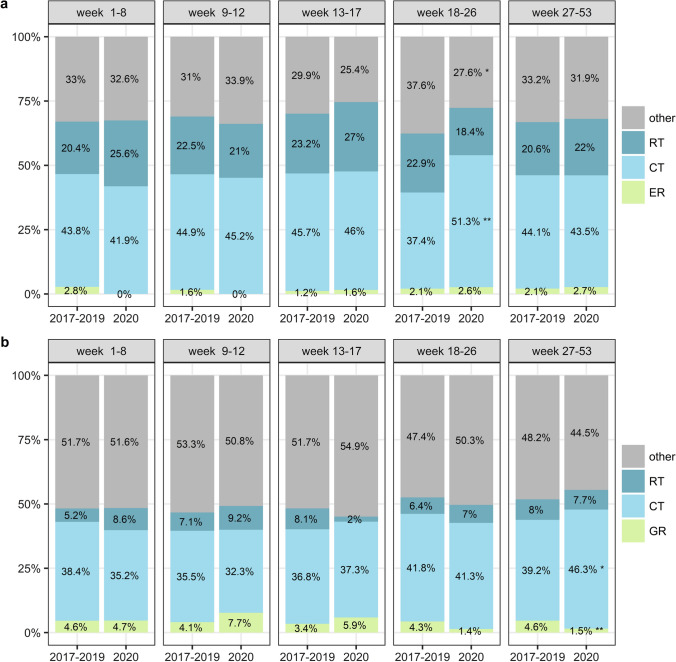
Plot of the overview of treatments given in the palliative setting stratified by period of diagnosis where **a** shows the treatments of patients with incurable esophageal cancer and **b** shows the treatments in patients with incurable gastric cancer. ER; esophageal resection with or without other treatments, GR; gastric resection with or without other treatments, CT; chemotherapy without resection and with or without other treatments, RT; radiotherapy without resection or chemotherapy

We observed a significant increase in the percentage of EC patients in the palliative setting treated with chemotherapy when healthcare was being upscaled in the Netherlands (period 4, from 37.4% in 2017–2019 to 51.3% in 2020, p = 0.004, Fig. 5a). At the same time, a decrease in the use of other treatments, such as best supportive care, was observed in this patient group (period 4, from 37.6% in 2017–2019 to 27.6% in 2020, *p* = 0.03, Fig. 5a*).* A similar pattern was seen in the group of GC patients in the palliative setting with relatively more patients being treated with chemotherapy in the second half of 2020 (period 5, from 39.2% in 2017–2019 to 46.3% in 2020, *p* = 0.02, Fig. 5b). Alternatively, in that same period, the percentage of patients with GC that underwent resection was significantly lower than in the years before (from 4.6% in 2017–2019 to 1.5% in 2020, *p* = 0.009, Fig. 5b). Over the whole year of 2020, no significant changes were found in the treatment of patients with incurable disease.

### Time intervals

The time to start treatment was significantly shorter during and after the COVID-19 outbreak in the Netherlands both in the potentially curative setting and in the palliative setting for EC (Table 2) and GC patients (Table 3). This was most prominent for GC patients with potentially curable disease just before the lockdown (period 2, from 6.9 weeks in 2017–2019 to 5.0 weeks in 2020, *p* = 0.04, Table 3). In contrast, the time between nCRT and resection increased after the lockdown for EC patients with potentially curable disease (in period 4 from 11.1 weeks in 2017–2019 to 12.3 weeks in 2020, *p* = 0.03 and in period 5 from 11.0 weeks in 2017–2019 to 12.3 weeks in 2020, *p* < 0.001). There was no difference in the time until direct resection for patients suffering from GC or EC in any of the periods in 2017–2019 as compared to 2020.

**Table 2:** Time between diagnosis and start treatment and time between neoadjuvant chemoradiotherapy and resection in esophageal cancer patients stratified by period

	Time from diagnosis to start treatment in patients with potentially curable disease			Time from diagnosis to start treatment in patients with incurable disease			Time from diagnosis to direct resection in patients with potentially curable disease			Time from end of nCRT to resection in patients with potentially curable disease		
	2017–2019	2020	*p*	2017–2019	2020	*p*	2017–2019	2020	*p*	2017–2019	2020	*p*
Week 1–8	6.18 (3.79)	6.00 (3.46)	0.539	5.00 (3.60)	5.20 (2.64)	0.635	9.68 (6.53)	8.54 (6.02)	0.766	11.17 (4.51)	10.12 (3.31)	0.058
Week 9–12	6.23 (3.70)	5.44 (2.60)	0.069	4.88 (3.37)	4.78 (4.10)	0.881	26.75 (13.31)	15.00 (21.21)	0.435	11.55 (5.09)	10.75 (4.47)	0.464
Week 13–17	6.74 (4.19)	5.28 (2.09)	0.001	4.87 (2.66)	4.46 (2.37)	0.339	11.08 (5.69)	7.43 (2.65)	0.320	11.82 (4.89)	11.65 (4.44)	0.848
Week 18–26	6.47 (5.27)	5.74 (2.81)	0.034	5.23 (2.82)	4.57 (2.29)	0.027	8.96 (3.68)	9.18 (6.12)	0.934	11.13 (3.76)	12.28 (5.16)	0.027
Week 27–53	6.32 (3.56)	5.95 (3.13)	0.017	4.91 (2.56)	5.05 (2.83)	0.418	12.05 (9.08)	11.34 (6.18)	0.809	10.95 (4.16)	12.32 (4.53)	< 0.001

Data for 2017–2019 are shown as the average of the 3 years. All data are reported in number of weeks, represented by mean (SD)

nCRT; neoadjuvant chemoradiotherapy

**Table 3:** Time between diagnosis and start treatment and time between neoadjuvant chemotherapy and resection in gastric cancer patients stratified by period

	Time from diagnosis to start treatment in patients with potentially curable disease			Time from diagnosis to start treatment in patients with incurable disease			Time from diagnosis to direct resection in patients with potentially curable disease			Time from end of neoadjuvant chemo to resection in patients with potentially curable disease		
	2017–2019	2020	*p*	2017–2019	2020	p	2017–2019	2020	*p*	2017–2019	2020	*p*
Week 1–8	6.60 (3.76)	6.23 (3.40)	0.397	5.14 (3.14)	5.86 (5.04)	0.186	7.85 (4.23)	7.18 (2.95)	0.500	7.31 (2.56)	6.57 (3.50)	0.128
Week 9–12	6.93 (4.40)	4.97 (3.92)	0.044	5.27 (6.29)	5.40 (3.23)	0.906	6.92 (3.31)	4.57 (4.75)	0.080	7.54 (3.40)	5.76 (1.48)	0.176
Week 13–17	7.48 (4.06)	6.22 (4.17)	0.116	5.63 (3.81)	4.96 (2.24)	0.416	9.33 (4.87)	6.56 (6.97)	0.170	7.50 (2.64)	7.59 (3.61)	0.914
Week 18–26	7.30 (5.12)	6.41 (2.98)	0.127	6.01 (4.84)	4.83 (3.52)	0.048	9.04 (6.24)	6.67 (3.55)	0.063	7.24 (2.51)	7.59 (2.76)	0.469
Week 27–53	7.22 (4.71)	6.62 (4.01)	0.060	5.50 (3.79)	4.86 (2.87)	0.020	7.96 (4.20)	7.54 (5.53)	0.505	7.24 (2.81)	7.36 (3.09)	0.671

Data for 2017–2019 are shown as the average of the 3 years. All data are reported in number of weeks, represented by mean (SD)

### Overall survival

EC patients diagnosed with incurable disease in the period after the lockdown had a significantly better 2-year overall survival compared to patients diagnosed in the same period in previous years (period 4, HR = 0.78, 95% CI 0.62–0.98, *p* = 0.04). GC patients diagnosed with curable disease in the period just before the lockdown had a significantly worse 2-year overall survival compared to patients diagnosed in the same period in the previous years (period 2, HR = 1.87, 95% CI 1.03–3.41, *p* = 0.04). There was no difference in 2-year overall survival for EC patients diagnosed with potentially curable disease or GC patients diagnosed with incurable disease in any of the periods or when comparing the whole year 2020 to 2017–2019 (*Online Resource 2*).

## Discussion

This analysis including data from all hospitals in the Netherlands describes the effect of the COVID-19 pandemic on the incidence, stage, and treatment of patients with esophagogastric cancer. We observed a profound decrease in the number of esophagogastric cancer diagnoses in the first 2 months of the COVID-19 pandemic. Comparable decreases in the incidence of esophagogastric cancer during the pandemic were observed in Japan, Italy, and France. [19–21] Similar to observations made in colorectal cancer, we observed that the decrease in number of GC diagnoses was fully compensated in the second half of 2020. [9] We suspect that reports of fewer cancer diagnoses in the Dutch media, and the call to patients to seek medical care may have boosted this catch-up effect. However, for unknown reasons, this catch-up did not fully compensate for the number of esophageal cancer diagnoses, resulting in a decreased incidence of 5.2% in 2020 compared to previous years. Potentially, the catch-up of EC diagnoses continued in 2021, but we performed a preliminary analysis that did not support this hypothesis.

Among the patients diagnosed during the first COVID-19 wave, a relatively high percentage was diagnosed with incurable gastric cancer, while a relatively low percentage was diagnosed with curable disease. A comparable decline in number of diagnoses and redistribution to higher disease stage was reported in the Netherlands for prostate cancer, breast cancer, and colorectal cancer [7–9]. Potentially, patients with relatively few and not alarming symptoms were less likely to visit a general practitioner during the start of the COVID-19 pandemic. This is supported by the findings of Lantinga et al. [22], who found a decrease in the total number of endoscopies and cancer diagnoses in that period but an increase in the percentage of patients undergoing an endoscopy in whom a malignancy was suspected. Although the percentage of patients diagnosed with curable disease was lower, detection of early carcinoma did not differ between 2017 and 2019 and 2020.

Similar to previous findings in colorectal cancer and across all cancer types, we found a significant impact of the COVID-19 pandemic on the type of treatment given both in the potentially curative setting and in the palliative setting for esophagogastric cancer patients [23, 24]. We detected a decrease in the number of gastric and esophageal resections 5months after the lockdown. This may very well have been a direct result of the decrease in the number of diagnoses during the lockdown, since the time between diagnosis and resection in these patients is often around 5 months. A possible explanation for the decrease in the number of resections could be the increase in the number of interval metastases in patients diagnosed during and after the social lockdown, resulting in an increase in neoadjuvant treatment without surgery.

Another possible explanation for the decrease of treatment with nCRT followed by resection compared to nCRT alone is an increase in wait-and-see approach. The wait-and-see approach was investigated in the Dutch Surgery As Needed for Oesophageal Cancer (SANO) trial, where EC patients with a clinical complete response after nCRT underwent active surveillance (and surgical resection only when residual tumor was found in the absence of distant metastases) [25]. The stepped-wedge design of this trial resulted in more patients being treated with nCRT alone in 2020 compared to the previous years. However, we would expect the effect to be significant in the entire year if the decrease in nCRT with resection would only be explained by the SANO trial. The fact that the decrease in resections was only significant for patients diagnosed during and after the lockdown suggests that the COVID-19 pandemic also impacted treatment. Possibly, there was a reluctance to perform surgical procedures during the pandemic, because these require resources that were redirected to care for COVID-19 patients. There are two separate observations that support this notion. First, we found a relative increase in patients treated with dCRT for patients diagnosed with potentially curable EC compared to nCRT with surgery. Second, we also observed a relative reduction in palliative gastric resections performed in the second half of 2020 for patients diagnosed with incurable GC. Of note, surgical care during the COVID-19 pandemic was not associated with an increase in postoperative complications, neither pulmonary nor other [26, 27].

The differences in treatment during the COVID-19 pandemic did partially influence survival. Patients diagnosed with incurable EC were treated more frequently with chemotherapy in the months after the social lockdown. A concurrent decrease in EC patients receiving other treatments such as best supportive care in the palliative setting was found. This shift in treatment toward chemotherapy resulted in a significantly better 2-year overall survival for EC patients in the palliative setting. As mentioned before, patients diagnosed with incurable GC in the second half of 2020 were less likely to undergo a palliative gastric resection. We observed a concurrent increase in the percentage of incurable GC patients treated with chemotherapy. This decrease in palliative resections did not impact overall survival, as a gastrectomy for advanced gastric cancer is known to have no added survival benefit [28]. For patients with potentially curable GC, there was a decreasing trend in CT-GR with a concurrent increase in GR alone after the first COVID-19 patient was diagnosed in the Netherlands. This increase in GR alone for potentially curable GC is also visible by the peak of GR in March. This shift toward GR alone instead of CT-GR is a possible explanation for the significantly poorer 2-year survival for patients with potentially curable GC diagnosed just before the lockdown. For potentially curable EC patients, there was a significant decrease in nCRT-ER in the second half of 2020 and a concurrent increase dCRT. In spite of this, there was no difference in 2-year overall survival. Treating esophageal cancer with nCRT followed by resection has a 2-year survival of 65% while treatment with dCRT has a 2-year overall survival of 55% [29, 30]. However, this difference increases over time, where nCRT with a resection has a 3-year survival of 58% while treatment with dCRT has a 3-year survival of 42%. Thus, with the current relatively short follow-up, long-term survival differences cannot be ruled out.

We also observed differences in the time to and between treatments. Interestingly, we found that the time between diagnosis and start of treatment was significantly shorter for most patients in 2020. Possibly the decrease in the number of patients diagnosed with esophagogastric cancer allowed for faster scheduling in the outpatient clinic. The clinical relevance of starting treatment 1 week sooner is debatable, as our results also show that overall survival was not different for almost all subpopulations.

While time between diagnosis and start of treatment was significantly shorter, time to surgery after neoadjuvant treatment was significantly longer in the second half of 2020. Since the effect of longer time to surgery was not present during the first half of 2020, it is again unlikely that the SANO trial is the only explanation for the increased time to surgery. Probably, scheduling difficulties occurred in the second half of 2020 due to upscaling of healthcare. In line with the existing literature, we found no impact on overall survival for a longer time to surgery after nCRT [31–33].

This study is the first to show the effect of the COVID-19 pandemic on the incidence, stage, and treatment of esophagogastric cancer. It allowed for a nationwide investigation of not only the incidence and stage of patients with esophagogastric cancer but also the types of treatment that were given. A limitation may be the relatively low number of reference years, which posed some statistical difficulties when comparing the absolute number of diagnoses and resections. We chose these reference years, since they were most comparable due to the changing epidemiology of esophagogastric cancer in the previous years [34]. This approach is also frequently used in other studies [7–9]. Another limitation is the relatively short follow-up data of 1–2 years. It will be interesting to analyze the association between the differences in treatment that are observed in this study and survival data with a longer follow-up.

In conclusion, the incidence of esophagogastric cancer diagnoses decreased significantly during the COVID-19 pandemic in the Netherlands. Relatively more incurable patients with worse performance scores were diagnosed. In addition, we found evidence that fewer surgical resections were performed.

### Supplementary Information

Below is the link to the electronic supplementary material.

**Supplementary file 1.**
