# Supplementary material for: The impact of the COVID-19 pandemic on the diagnosis, stage, and treatment of esophagogastric cancer
Source: J Gastroenterol. 2023 Jul 31;58(10):965–77. doi: 10.1007/s00535-023-02009-3 (PMC10522512; doi:10.1007/s00535-023-02009-3)
Supplement: Supplementary file 1 [file 535_2023_2009_MOESM1_ESM.docx]

**Supplementary Material for Journal of Gastroenterology**

**The impact of the COVID-19 pandemic on the diagnosis, stage, and treatment of esophagogastric cancer**

Benthe H. Doeve^1,2,3,4^°, Jeanne A.C. Bakx^1,2,5^°, Peter D. Siersema^6^, Camiel Rosman^7^, Nicole C.T. van Grieken^2,8^, Mark I. van Berge Henegouwen^2,9^, Johanna W. van Sandick^10^, Marcel Verheij^11^, Maarten F. Bijlsma^2,3,4^, Rob H.A. Verhoeven^1.5*^, Hanneke W.M. van Laarhoven^1,2*^

^1^ Amsterdam UMC, location University of Amsterdam, Department of Medical Oncology, Meibergdreef 9, Amsterdam, The Netherlands

^2^ Cancer Center Amsterdam, Cancer Treatment and Quality of Life, Amsterdam, The Netherlands

^3^ Amsterdam UMC, University of Amsterdam, Center for Experimental and Molecular Medicine, Laboratory for Experimental Oncology and Radiobiology, Meibergdreef 9, Amsterdam, The Netherlands

^4^ Oncode Institute, Meibergdreef 9, Amsterdam, The Netherlands

^5^ Department of Research and Development, Netherlands Comprehensive Cancer Organisation (IKNL), Utrecht, The Netherlands.

^6^ Department of Gastroenterology and Hepatology | Erasmus MC University Medical Center | Rotterdam, The Netherlands.

^7^ Department of Surgery, Radboud University Medical Center, Nijmegen 6500 HB, The Netherlands

^8^ Amsterdam UMC, location Vrije Universteit Amsterdam, Department of Pathology, De Boelelaan 1117, Amsterdam, The Netherlands

^9^ Amsterdam UMC, location University of Amsterdam, Department of Surgery, Meibergdreef 9, Amsterdam, The Netherlands

^10^ Department of Surgery, Antoni van Leeuwenhoek Hospital - Netherlands Cancer Institute, Amsterdam, The Netherlands

^11^ Department of Radiation Oncology, Radboud University Medical Center, Nijmegen 6500 HB, The Netherlands

° Share first authorship

* Share senior authorship

Corresponding author: Mrs. B.H. Doeve, De Boelelaan 1118, 1081 HZ Amsterdam, The Netherlands, Email: [b.h.doeve@amsterdamumc.nl](mailto:b.h.doeve@amsterdamumc.nl)

**Online Resource 1**

| **Supplementary table 1A** Baseline characteristics of patients with esophageal cancer, stratified by period and year of diagnosis. | | | | | | | | | | | | | | | |
| --- | --- | --- | --- | --- | --- | --- | --- | --- | --- | --- | --- | --- | --- | --- | --- |
|  | **WEEK 1-8** | | | **WEEK 9-12** | | | **WEEK 13-17** | | | **WEEK 18-26** | | | **WEEK 27-53** | | |
|  | *2017-2019* | *2020* | *p* | *2017-2019* | *2020* | *p* | *2017-2019* | *2020* | *p* | *2017-2019* | *2020* | *p* | *2017-2019* | *2020* | *p* |
| **Patients** | **1204** | **391** |  | **566** | **152** |  | **719** | **180** |  | **1287** | **438** |  | **3792** | **1230** |  |
| **Sex** |  |  |  |  |  |  |  |  |  |  |  |  |  |  |  |
| Female | 335 (27.8) | 121 (30.9) | 0.262 | 125 (22.1) | 40 (26.3) | 0.321 | 189 (26.3) | 45 (25.0) | 0.797 | 338 (26.3) | 120 (27.4) | 0.688 | 958 (25.3) | 338 (27.5) | 0.132 |
| Male | 869 (72.2) | 270 (69.1) |  | 441 (77.9) | 112 (73.7) |  | 530 (73.7) | 135 (75.0) |  | 949 (73.7) | 318 (72.6) |  | 2834 (74.7) | 892 (72.5) |  |
| **Age** |  |  |  |  |  |  |  |  |  |  |  |  |  |  |  |
| <60 years | 203 (16.9) | 50 (12.8) | 0.144 | 91 (16.1) | 30 (19.7) | 0.563 | 111 (15.4) | 27 (15.0) | 0.981 | 189 (14.7) | 72 (16.4) | 0.212 | 614 (16.2) | 188 (15.3) | 0.632 |
| 60-74 years | 630 (52.3) | 210 (53.7) |  | 298 (52.7) | 77 (50.7) |  | 394 (54.8) | 100 (55.6) |  | 703 (54.6) | 218 (49.8) |  | 1953 (51.5) | 651 (52.9) |  |
| >74 years | 371 (30.8) | 131 (33.5) |  | 177 (31.3) | 45 (29.6) |  | 214 (29.8) | 53 (29.4) |  | 395 (30.7) | 148 (33.8) |  | 1225 (32.3) | 391 (31.8) |  |
| **Histology** |  |  |  |  |  |  |  |  |  |  |  |  |  |  |  |
| AC | 890 (73.9) | 290 (74.2) | 0.993 | 433 (76.5) | 114 (75.0) | 0.506 | 539 (75.0) | 126 (70.0) | 0.202 | 925 (71.9) | 325 (74.2) | 0.500 | 2755 (72.7) | 883 (71.8) | 0.429 |
| SCC | 302 (25.1) | 97 (24.8) |  | 129 (22.8) | 38 (25.0) |  | 176 (24.5) | 54 (30.0) |  | 351 (27.3) | 111 (25.3) |  | 1008 (26.6) | 333 (27.1) |  |
| Other | 12 (1.0) | 4 (1.0) |  | 4 (0.7) | 0 (0.0) |  | 4 (0.6) | 0 (0.0) |  | 11 (0.9) | 2 (0.5) |  | 29 (0.8) | 14 (1.1) |  |
| **Stage** |  |  |  |  |  |  |  |  |  |  |  |  |  |  |  |
| Early carcinoma | 37 (3.1) | 11 (2.8) | 0.966 | 16 (2.8) | 5 (3.3) | 0.177 | 27 (3.8) | 4 (2.2) | 0.587 | 32 (2.5) | 10 (2.3) | 0.932 | 95 (2.5) | 37 (3.0) | 0.136 |
| Potentially curable | 767 (63.9) | 250 (64.1) |  | 363 (64.1) | 85 (55.9) |  | 438 (60.9) | 113 (62.8) |  | 819 (63.6) | 276 (63.0) |  | 2396 (63.2) | 738 (60.0) |  |
| Incurable | 397 (33.1) | 129 (33.1) |  | 187 (33.0) | 62 (40.8) |  | 254 (35.3) | 63 (35.0) |  | 436 (33.9) | 152 (34.7) |  | 1298 (34.2) | 451 (36.7) |  |
| **Comorbidities** |  |  |  |  |  |  |  |  |  |  |  |  |  |  |  |
| 0 | 504 (44.2) | 162 (42.9) | 0.794 | 227 (42.2) | 71 (48.0) | 0.293 | 291 (43.0) | 77 (43.3) | 0.997 | 521 (42.4) | 191 (44.0) | 0.812 | 1660 (46.1) | 555 (45.8) | 0.611 |
| 1 | 387 (33.9) | 127 (33.6) |  | 175 (32.5) | 48 (32.4) |  | 236 (34.9) | 62 (34.8) |  | 405 (32.9) | 141 (32.5) |  | 1157 (32.1) | 377 (31.1) |  |
| 2 or more | 250 (21.9) | 89 (23.5) |  | 136 (25.3) | 29 (19.6) |  | 150 (22.2) | 39 (21.9) |  | 304 (24.7) | 102 (23.5) |  | 783 (21.8) | 279 (23.0) |  |
| **Performance status** |  |  |  |  |  |  |  |  |  |  |  |  |  |  |  |
| ECOG 0 | 373 (41.9) | 114 (36.7) | **0.018** | 150 (36.7) | 39 (34.2) | 0.900 | 225 (41.2) | 53 (37.3) | 0.801 | 354 (35.9) | 150 (41.0) | 0.152 | 1120 (38.8) | 385 (38.3) | 0.189 |
| ECOG 1 | 354 (39.7) | 114 (36.7) |  | 175 (42.8) | 50 (43.9) |  | 215 (39.4) | 57 (40.1) |  | 413 (41.9) | 129 (35.2) |  | 1212 (42.0) | 414 (41.2) |  |
| ECOG 2 | 94 (10.5) | 50 (16.1) |  | 59 (14.4) | 19 (16.7) |  | 74 (13.6) | 22 (15.5) |  | 145 (14.7) | 60 (16.4) |  | 373 (12.9) | 122 (12.1) |  |
| ECOG 3 or 4 | 70 (7.9) | 33 (10.6) |  | 25 (6.1) | 6 (5.3) |  | 32 (5.9) | 10 (7.0) |  | 74 (7.5) | 27 (7.4) |  | 184 (6.4) | 84 (8.4) |  |
| All data are represented as n (%). Counts for 2017-2019 are shown as the sum of the three years. Percentages for 2017-2019 are shown as the average of the three years. | | | | | | | | | | | | | | | |

| **Supplementary table 1B** Baseline characteristics of patients with gastric cancer, stratified by period and year of diagnosis. | | | | | | | | | | | | | | | |
| --- | --- | --- | --- | --- | --- | --- | --- | --- | --- | --- | --- | --- | --- | --- | --- |
|  | **WEEK 1-8** | | | **WEEK 9-12** | | | **WEEK 13-17** | | | **WEEK 18-26** | | | **WEEK 27-53** | | |
|  | *2017-2019* | *2020* | *p* | *2017-2019* | *2020* | *p* | *2017-2019* | *2020* | *p* | *2017-2019* | *2020* | *p* | *2017-2019* | *2020* | *p* |
| **Patients** | **683** | **255** |  | **375** | **96** |  | **416** | **95** |  | **759** | **256** |  | **2092** | **729** |  |
| **Sex** |  |  |  |  |  |  |  |  |  |  |  |  |  |  |  |
| Female | 239 (35.0) | 87 (34.1) | 0.862 | 149 (39.7) | 38 (39.6) | 1.000 | 141 (33.9) | 41 (43.2) | 0.114 | 273 (36.0) | 94 (36.7) | 0.888 | 747 (35.7) | 255 (35.0) | 0.757 |
| Male | 444 (65.0) | 168 (65.9) |  | 226 (60.3) | 58 (60.4) |  | 275 (66.1) | 54 (56.8) |  | 486 (64.0) | 162 (63.3) |  | 1345 (64.3) | 474 (65.0) |  |
| **Age** |  |  |  |  |  |  |  |  |  |  |  |  |  |  |  |
| <60 years | 124 (18.2) | 45 (17.6) | 0.983 | 57 (15.2) | 17 (17.7) | 0.834 | 67 (16.1) | 16 (16.8) | 0.655 | 148 (19.5) | 39 (15.2) | 0.100 | 346 (16.5) | 155 (21.3) | **0.012** |
| 60-74 years | 265 (38.8) | 100 (39.2) |  | 149 (39.7) | 37 (38.5) |  | 144 (34.6) | 37 (38.9) |  | 295 (38.9) | 92 (35.9) |  | 825 (39.4) | 261 (35.8) |  |
| >74 years | 294 (43.0) | 110 (43.1) |  | 169 (45.1) | 42 (43.8) |  | 205 (49.3) | 42 (44.2) |  | 316 (41.6) | 125 (48.8) |  | 921 (44.0) | 313 (42.9) |  |
| **Histology** |  |  |  |  |  |  |  |  |  |  |  |  |  |  |  |
| AC | 674 (98.7) | 251 (98.4) | 1.000 | 367 (97.9) | 95 (99.0) | 0.780 | 413 (99.3) | 94 (98.9) | 1.000 | 755 (99.5) | 252 (98.4) | 0.226 | 2072 (99.0) | 718 (98.5) | 0.304 |
| Other | 9 (1.3) | 4 (1.6) |  | 8 (2.1) | 1 (1.0) |  | 3 (0.7) | 1 (1.1) |  | 4 (0.5) | 4 (1.6) |  | 20 (1.0) | 11 (1.5) |  |
| **Stage** |  |  |  |  |  |  |  |  |  |  |  |  |  |  |  |
| Early carcinoma | 16 (2.3) | 9 (3.5) | 0.604 | 13 (3.5) | 1 (1.0) | **0.022** | 11 (2.6) | 0 (0.0) | 0.212 | 18 (2.4) | 4 (1.6) | 0.441 | 36 (1.7) | 18 (2.5) | 0.225 |
| Potentially curable | 321 (47.0) | 118 (46.3) |  | 165 (44.0) | 30 (31.2) |  | 171 (41.1) | 44 (46.3) |  | 347 (45.8) | 109 (42.6) |  | 977 (46.7) | 320 (43.9) |  |
| Incurable | 346 (50.7) | 128 (50.2) |  | 197 (52.5) | 65 (67.7) |  | 234 (56.2) | 51 (53.7) |  | 392 (51.8) | 143 (55.9) |  | 1077 (51.5) | 391 (53.6) |  |
| **Comorbidities** |  |  |  |  |  |  |  |  |  |  |  |  |  |  |  |
| 0 | 292 (45.5) | 110 (44.5) | 0.795 | 141 (40.2) | 43 (45.3) | 0.530 | 173 (44.7) | 33 (34.7) | 0.105 | 328 (45.6) | 107 (42.1) | 0.460 | 856 (43.7) | 332 (46.1) | 0.505 |
| 1 | 196 (30.5) | 81 (32.8) |  | 122 (34.8) | 33 (34.7) |  | 128 (33.1) | 42 (44.2) |  | 231 (32.1) | 81 (31.9) |  | 649 (33.1) | 231 (32.1) |  |
| 2 or more | 154 (24.0) | 56 (22.7) |  | 88 (25.1) | 19 (20.0) |  | 86 (22.2) | 20 (21.1) |  | 161 (22.4) | 66 (26.0) |  | 456 (23.3) | 157 (21.8) |  |
| **Performance status** |  |  |  |  |  |  |  |  |  |  |  |  |  |  |  |
| ECOG 0 | 152 (35.6) | 60 (35.3) | 0.987 | 75 (30.5) | 21 (32.3) | 0.992 | 91 (33.5) | 20 (30.8) | 0.234 | 174 (35.4) | 64 (34.8) | **0.018** | 466 (33.8) | 209 (37.3) | 0.170 |
| ECOG 1 | 165 (38.6) | 67 (39.4) |  | 99 (40.2) | 26 (40.0) |  | 105 (38.6) | 19 (29.2) |  | 220 (44.7) | 65 (35.3) |  | 540 (39.1) | 189 (33.8) |  |
| ECOG 2 | 62 (14.5) | 23 (13.5) |  | 40 (16.3) | 10 (15.4) |  | 42 (15.4) | 16 (24.6) |  | 57 (11.6) | 27 (14.7) |  | 227 (16.4) | 97 (17.3) |  |
| ECOG 3 or 4 | 48 (11.2) | 20 (11.8) |  | 32 (13.0) | 8 (12.3) |  | 34 (12.5) | 10 (15.4) |  | 41 (8.3) | 28 (15.2) |  | 147 (10.7) | 65 (11.6) |  |
| All data are represented as n (%). Counts for 2017-2019 are shown as the sum of the three years. Percentages for 2017-2019 are shown as the average of the three years. | | | | | | | | | | | | | | | |

**Online Resource 2**

| **Supplementary table 2** Two-year overall survival of patients diagnosed in 2020 compared to patients diagnosed in 2017-2019, stratified by period of diagnosis | | | | | |
| --- | --- | --- | --- | --- | --- |
|  |  | **Period** | **HR** | **95% CI** | **p-value** |
| Potentially curable EC |  | 1 | 1.05 | 0.83 - 1.32 | 0.704 |
|  |  | 2 | 1.23 | 0.85 - 1.78 | 0.269 |
|  |  | 3 | 0.92 | 0.65 - 1.31 | 0.662 |
|  |  | 4 | 1.06 | 0.85 - 1.33 | 0.606 |
|  |  | 5 | 1.06 | 0.91 - 1.22 | 0.456 |
|  |  | Complete year | 1.05 | 0.96 - 1.16 | 0.298 |
| Potentially curable GC |  | 1 | 1.03 | 0.72 - 1.49 | 0.860 |
|  |  | 2 | 1.87 | 1.03 - 3.41 | **0.040** |
|  |  | 3 | 1.01 | 0.58 - 1.78 | 0.967 |
|  |  | 4 | 0.92 | 0.61 - 1.38 | 0.676 |
|  |  | 5 | 0.83 | 0.65 - 1.05 | 0.120 |
|  |  | Complete year | 0.96 | 0.86 - 1.12 | 0.598 |
| Incurable EC |  | 1 | 1.01 | 0.79 - 1.30 | 0.931 |
|  |  | 2 | 1.06 | 0.74 - 1.52 | 0.749 |
|  |  | 3 | 0.96 | 0.66 - 1.40 | 0.838 |
|  |  | 4 | 0.78 | 0.62 – 0.98 | **0.036** |
|  |  | 5 | 1.07 | 0.93 - 1.22 | 0.332 |
|  |  | Complete year | 0.97 | 0.89 - 1.07 | 0.600 |
| Incurable GC |  | 1 | 0.98 | 0.75 - 1.28 | 0.888 |
|  |  | 2 | 1.19 | 0.82 - 1.73 | 0.370 |
|  |  | 3 | 0.68 | 0.44 - 1.05 | 0.083 |
|  |  | 4 | 1.10 | 0.86 - 1.41 | 0.457 |
|  |  | 5 | 1.08 | 0.93 - 1.26 | 0.317 |
|  |  | Complete year | 1.03 | 0.92 - 1.14 | 0.631 |
| 2-year survival stratified by year of diagnosis, where the 2017-2019 was used as the reference value. Corrected for sex, age, comorbidities and performance status.  HR = Hazard Ratio, CI = Confidence Interval | | | | | |
